# Integrated cost-benefit analysis of tsetse control and herd productivity to inform control programs for animal African trypanosomiasis

**DOI:** 10.1186/s13071-018-2679-x

**Published:** 2018-03-07

**Authors:** Anne Meyer, Hannah R. Holt, Farikou Oumarou, Kalinga Chilongo, William Gilbert, Albane Fauron, Chisoni Mumba, Javier Guitian

**Affiliations:** 1 Department of Pathobiology and Population Sciences, Royal Veterinary College, Hatfield, UK; 2 Mission Spéciale d’Eradication des Glossines, Ngaoundéré, Cameroon; 3 Department of Veterinary Services, Tsetse and Trypanosomiasis Control Unit, Ministry of Fisheries and Livestock, Lusaka, Zambia; 4 Department of Disease Control, School of Veterinary Medicine, University of Zambia, P.O. Box 32379, Lusaka, Zambia

**Keywords:** Trypanosomiasis, Sub-Saharan Africa, Cameroon, Zambia, Cost-benefit analysis, Vector control, Tsetse, Cattle, Bio-economic model

## Abstract

**Background:**

Animal African trypanosomiasis (AAT) and its tsetse vector are responsible for annual losses estimated in billions of US dollars ($). Recent years have seen the implementation of a series of multinational interventions. However, actors of AAT control face complex resource allocation decisions due to the geographical range of AAT, diversity of ecological and livestock systems, and range of control methods available.

**Methods:**

The study presented here integrates an existing tsetse abundance model with a bio-economic herd model that captures local production characteristics as well as heterogeneities in AAT incidence and breed. These models were used to predict the impact of tsetse elimination on the net value of cattle production in the districts of Mambwe, in Zambia, and Faro et Déo in Cameroon. The net value of cattle production under the current situation was used as a baseline, and compared with alternative publicly funded control programmes. In Zambia, the current baseline is AAT control implemented privately by cattle owners (Scenario Z0). In Cameroon, the baseline (Scenario C0) is a small-scale publicly funded tsetse control programme and privately funded control at farm level. The model was run for 10 years, using a discount rate of 5%.

**Results:**

Compared to Scenario C0, benefit-cost ratios (BCR) of 4.5 (4.4–4.7) for Scenario C1 (tsetse suppression using insecticide treatment of cattle (ITC) and traps + maintenance with ITC barrier), and 3.8 (3.6–4.0) for Scenario C2 (tsetse suppression using ITC and traps + maintenance with barrier of targets), were estimated in Cameroon. For Zambia, the benefit-cost ratio calculated for Scenarios Z1 (targets, ITC barrier), Z2 (targets, barrier traps), Z3 (aerial spraying, ITC barrier), and Z4 (aerial spraying, barrier traps) were 2.3 (1.8 - 2.7), 2.0 (1.6-2.4), 2.8 (2.3–3.3) and 2.5 (2.0–2.9), respectively. Sensitivity analysis showed that the profitability of the projects is relatively resistant to variations in the costs of the interventions and their technical efficiency.

**Conclusions:**

It is envisioned that the methodologies presented here will be useful for the evaluation and design of existing and future control programmes, ensuring they have tangible benefits in the communities they are targeting.

**Electronic supplementary material:**

The online version of this article (10.1186/s13071-018-2679-x) contains supplementary material, which is available to authorized users.

## Background

According to 2013 estimates, half of the world’s poor live in sub-Saharan Africa (World Bank, 2017) and 63% of them are located in rural areas. Ensuring food security in rural areas of sub-Saharan Africa is an important challenge, and sustainable development of agriculture is recognised as a key strategy for poverty reduction [1]. Animal African trypanosomiasis (AAT) is a devastating livestock disease, responsible for total annual losses estimated in the billions of dollars (US$) in sub-Saharan Africa [2, 3]. The disease is caused by a parasite protozoan of the genus *Trypanosoma*; its primary vector, tsetse flies (*Glossina* spp.), infests around 10 million km^2^ of sub-Saharan Africa [4]. AAT affects the health and productivity of livestock to the extent that it influences where people settle, as well as the intensity and diversity of both crop and livestock industries [5]. The impact of AAT itself can be reduced by curative and prophylactic trypanocide applications and the breeding of trypanotolerant cattle. However, there is increasing resistance to trypanocides [6, 7] and farmers are often reluctant to use trypanotolerant breeds [8]. Vaccines are unavailable, and reduction of transmission relies on the control of the tsetse vectors by insecticide treatment of cattle (ITC), the use of traps or insecticide-treated targets (ITT), ground or aerial insecticide spraying, and reducing the risk of exposure to the vector through changes in livestock management. In addition to AAT, vector control plays a major role in combatting Rhodesian human African trypanosomiasis (HAT) [9], for which cattle are an important reservoir [10], and contributes to global efforts against Gambian HAT (caused by *T. brucei gambiense*) [11]. The most appropriate tools for control and the scale at which to implement them depend on the socio-economic and political context, physical environment, the eco-epidemiological cycle of AAT, tsetse demographics and available resources [12].

Over the last 15 years, the Pan African Tsetse and Trypanosomiasis Eradication Campaign (PATTEC), established by the African Union, has supported a series of multinational interventions to control AAT and HAT in partnership with national governments [13]. Within this campaign, the African Development Bank has directed 72 million US$ in loans and grants to support the creation of tsetse-free areas in sub-Saharan African countries [13]. Despite this revitalised atmosphere of international co-operation and opportunity, decisions over the allocation of limited resources are still difficult, due to the enormous geographical range of the disease, the diversity of ecological and livestock systems within that range, and the variety of disease and vector control methods available. Hence, there is a need for transparent frameworks and tools for priority setting and resource allocation for future AAT control programmes [14, 15]. Predictions of the efficiency of resource allocation under different control scenarios using techniques such as cost-benefit analyses would be highly valuable in the planning of future programmes. For instance, selected macro-level economic evaluations provide important information for area prioritisation and the planning of AAT control programmes at regional level [16–18]. However, a disadvantage of such approaches is that aggregated indicators may overlook important heterogeneities at a small scale. Although area-wide tsetse elimination approaches have been advocated for over a decade [19–21], they require intensive planning, coordination and funding. Fifteen years after the launch of the PATTEC campaign, elimination activities are restricted to limited areas (Deme Valley in Ethiopia and Niayes area in Senegal), and in the absence of renewed funding opportunities, future large-scale elimination campaigns are uncertain. Local government services still play an important role in AAT control [22], but they are constrained by limited funds, infrastructure and human resources. A model that aids understanding of disease dynamics at a local level is therefore needed, to ensure choices of operational areas and control tools to maximise returns on investments.

Adapting previous work by Shaw et al. [12, 18, 23], this study proposes a framework for conducting a cost-benefit analysis of possible AAT control interventions in an administrative area, capturing within-population heterogeneities in term of disease incidence and livestock productivity. In this paper, we focus on AAT in cattle only. For illustration, we applied the framework to the Faro et Déo district of Cameroon and the Mambwe District of Zambia. Livestock owners in these areas rely heavily on chemotherapy and chemoprophylaxis, but with increasing levels of trypanocide resistance being reported [6, 24], both districts have been proposed as potential targets for coordinated tsetse and trypanosomiasis (T&T) control in their respective countries.

## Methods

### Conceptual framework for cost-benefit analysis

**Fig. 1:**
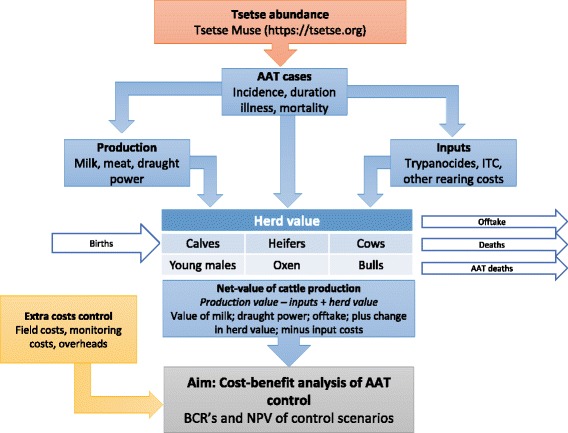
Schematic showing the different components considered in the cost-benefit analysis: (i) tsetse abundance (orange) and (ii) control costs (yellow) generated as a function of control programme, and (iii) herd model (blue) which generates herd value as a function of baseline production and tsetse abundance

A cost-benefit analysis was performed to estimate the financial returns of several potential AAT control strategies at district-level. First, detailed costs of the control interventions were estimated for the study area using existing country budgets and previous literature. Changes in tsetse abundance as a result of the interventions were then predicted using a vector abundance model, previously used by Shaw et al. [25]. These results were combined with estimates of the impact of AAT on cattle production in a bio-economic herd-model, to predict the net-value of cattle production in the study area under different interventions and baseline scenarios (Fig. 1). Additional revenues included in the model were: increased live weight, milk yield, draught power and calving rate as well as reduced mortality. A reduction in the incidence of disease resulted in costs saved due to reduced preventative and therapeutic action taken by farmers; in addition, the extra value of a larger herd under the AAT control scenarios was calculated [12, 26]. Extra costs (costs of implementing the control interventions) and revenue foregone (revenue lost under AAT control, i.e. reduction in salvage slaughter) as a result of the interventions were also compared to the baseline scenarios. Possible external factors such as changes in crop output, changes in pressure on natural resources and changes to herd management were not included. The total costs and benefits were estimated on an annual basis and totalled for each Scenario over a 10-year period using a discount rate of 5%. The economic performance indicators net present value (NPV) and the benefit-cost ratio (BCR) were then calculated.

### Study areas and scenarios considered

#### Cameroon case study

The Faro et Déo District of Cameroon is part of the most important cattle-producing region in the country (Adamaoua Region), supplying both local and international markets [27]. *Glossina morsitans submorsitans*, *Glossina fuscipes fuscipes* and *Glossina tachinoides* are the main tsetse species present. Despite a long history of tsetse control in the district [28], a cross-sectional study in 2010 estimated an overall AAT prevalence of up to 40.7%, thus still representing a significant threat to cattle production [29]. Currently, the district is divided into three zones: namely, the plateau, where tsetse populations have been suppressed, the buffer zone, used as a barrier to tsetse invasion from the valley to the plateau, and the tsetse-infested valley [27]. The current T&T control activities in place in the district (“Scenario C0”) include the regular use of trypanocides by farmers and small-scale control activities ran by the Mission Spéciale d’Eradication des Glossines (MSEG), which is part of the Department of Veterinary Services. The MSEG maintains a limited number of traps and targets, primarily in the buffer zone and carries out intermittent ground spraying. This is supplemented with some prophylactic trypanocide treatment and ITC for transhumant cattle moving between the tsetse-infested valley and the tsetse-free plateau. To inform the allocation of future funding, two alternative scenarios modelled the expansion of the current control activities into the infested zone (Scenarios C1 and C2). As the tsetse population is not isolated, a barrier would be required to prevent reinvasion from the Faro Game Park in the North and the Nigerian Gashaga Forest Reserve in the West. Scenarios C1 and C2 differed by the implementation of a barrier using ITC and ITT, respectively.

#### Zambia case study

The Mambwe District is part of the Luangwa Valley in the Eastern Province of Zambia, which has very high tsetse densities due to favourable vegetation and climate as well as an abundance of wildlife hosts [30]. Around three-quarters of this district is infested by tsetse, with *Glossina morsitans morsitans* being the main species present [31] and *Glossina pallidipes* also of epidemiological importance. The third species in the area, *Glossina brevipalpis,* is considered insignificant as a vector. The current baseline situation (“Scenario Z0”) consists of T&T control implemented by cattle owners only, who use a combination of insecticides and prophylactic and curative trypanocides. A study in a neighbouring district (Katete) revealed that 99% of the trypanocides were purchased indirectly from veterinary camps and district veterinary offices [32]. Four alternative local tsetse elimination campaigns under consideration for implementation by the Tsetse and Trypanosomiasis Control Unit (TTCU) of the Department of Veterinary Services were modelled for this district: two based on ITT (Scenarios Z1 and Z2) and two based on sequential aerial spraying (SAT) (Scenarios Z3 and Z4). As the current cattle density in Mambwe is low (2.4 head per km^2^), the use of ITC was not considered as an appropriate option for these settings. However, it might be used as a barrier, based on our model of cattle demographics. Therefore, implementation of a barrier to prevent tsetse reinvasion from the neighbouring South Luangwa National Park was accounted for, using ITC (Scenarios Z1 and Z3) and ITT (Scenarios Z2 and Z4), respectively.

### Implementing the control scenarios

#### Costs included in the different scenarios

The costs of implementing the control interventions were divided between administrative overheads, field costs and monitoring costs. The latter costs included initial entomological and parasitological surveys as well as subsequent T&T monitoring activities, all of which varied in relation to the level of the control activity. Budgets from the current control programme ran by the MSEG in Cameroon were consulted to obtain the costs of the current control programme (Scenario C0). To estimate the costs incurred by scenarios C1 and C2, current costs were extrapolated to the level of activity required to achieve tsetse elimination in the area. The control costs for the Mambwe District were inferred from the budget of previous control activities conducted by the TTCU in the Western Province of Zambia and complemented with data from the literature where necessary [23]. The detailed costs used in the calculations are available as supplementary material (Additional file 1: Table S1). In our simulation, ITC was applied at a density of four adult cattle per km^2^, as previous work predicted that local elimination would be achieved when applied at this density [33]. Although cattle density was lower than this cut-off in Mambwe at the start of the control programme, this is likely to increase once tsetse is suppressed in the district. The use of insecticide-impregnated, odour-baited targets was planned during the attack phase of Scenarios C1, C2, Z1 and Z2. This type of artificial baits has been used effectively in the past to eliminate savannah species of tsetse, for instance in Zambia and Zimbabwe [34]. The density of targets effectively used to eliminate savannah tsetse ranged from three to five per km^2^ in previous trials [35–38]. A density of four targets per km^2^ was chosen in this study. Scenarios Z3 and Z4 were based on the use of five cycles of SAT, as successfully used in neighbouring Botswana [39]. The barrier sizes were set as 8 km wide by 240 km long, and 8 km wide by 85 km long, in Faro et Déo and Mambwe, respectively, as recommended by previous work [35].

#### Simulating control scenarios

The interventions were split into three phases: (i) preparation phase when T&T surveys and awareness campaigns are conducted, and staff is recruited and trained; (ii) attack phase, when the interventions to eliminate the tsetse population in the area are deployed; and (iii) maintenance phase, when the barriers are maintained to prevent re-invasion from the adjacent areas. As a dedicated T&T unit already operates in the Faro et Déo District, conducting entomological surveys and engaging with farmers, no preparatory phase was considered for this study area. The publicly available Tsetse Muse model (http://www.tsetse.org/muse [36]) was used to predict the likely reduction in tsetse density under each scenario. Based on tsetse surveys on the Adamaoua plateau, a non-isolated starting population of 2500 females per km^2^ and 80 males per 100 females was simulated [37]. Birth, deaths, age structure and kill rates were set to recommended values [36]. The control techniques planned for each scenario and described above were used as inputs to run the tsetse abundance model. The time to eliminate tsetse predicted by the Tsetse Muse simulations was used to define the duration of the different scenario phases, allowing for additional time to accommodate potential delays and logistical issues. The duration of each phase in the different scenarios and the timing of the different control costs are shown in Table 1. Barriers would be set-up at the beginning of the attack phase, as this proved successful in a similar campaign in Botswana [39].

**Table 1:** Timing of the different T&T control scenarios in relation to the additional costs

Intervention phase	Preparation phase	Attack phase		Maintenance phase	
Scenario	No. of years	No. of years	Technique (elimination)	No. of years	Technique (barrier)
C1	0	2	ITC and targets	8	ITC
C2					Targets
Z1	2	2	Targets	6	ITC
Z2					Targets
Z3	2	1	SAT	7	ITC
Z4					Targets
Costs incurred	Overheads + monitoring costs	Overheads + attack field costs + barrier field costs + monitoring costs		Overheads + barrier field costs + monitoring costs	

*Abbreviations*: *ITC* insecticide treatment of cattle, *SAT*, sequential aerial treatment

### Estimating AAT incidence under the control scenarios

The annual incidence, defined as the cumulative number of AAT events in a given year out of the total cattle population at risk, varied according to the stage in the control programme and the zone within the study area where the control program is implemented. Three zones were considered in the Faro et Déo District based on different risks of AAT [27]. Estimates of the current annual incidence in the different zones of Faro et Déo were extracted from a year-long study conducted in sedentary herds in 2004 [6]. For transhumant herds, these data were combined with a prevalence study conducted in the valley between October and December 2005, which is when transhumant cattle enter the valley [38]. Over 1-year, the mean cumulative incidence values for sedentary herds on the plateau, sedentary herds in the valley and transhumant herds were 6.5, 53 and 22.6%, respectively. In Faro et Déo, 90% of the herds in the district were considered transhumant in the dry season, when the tsetse burden is at its lowest, while 10% were sedentary: of these, 2.5% resided in the valley, and 7.5% resided in the plateau (MSEG data). In the case of Zambia, the AAT incidence was considered to be homogenous in the infested area of the Mambwe District, which represents 80% of the total surface of the district (TTCU). During a longitudinal study in the Eastern Province of Zambia, 155 new infections were detected in 85 sentinel cattle over a period of 19 months [40]. The monthly incidence of AAT in cattle was estimated at 6 and 10% in two studies in the area [32, 40]. It was assumed therefore that the current annual incidence of AAT in Mambwe District ranged between 72 and 100%.

Based on AAT incidence surveys conducted in areas where tsetse had been eliminated, we considered that post-elimination AAT annual incidence in the different scenarios would lie between 1% and 5% [15, 28, 41–43]. Under the baseline scenarios (C0 and Z0), we considered that the current annual incidence of AAT in each district would remain stable. Under the control scenarios (C1 and C2, Z1 to Z4), we considered that the current AAT incidence would decrease proportionally with the decrease in tsetse density predicted by the vector abundance model, to reach the post-elimination value at the end of the attack phase.

### Bio-economic herd model

An animal-level herd model was developed to simulate the demographic, production and disease events within the cattle herd in the study area on an annual basis. The model included six age-sex cattle classes (calf, heifer, young male, cow, adult ox and adult bull) and accounted for the use of two different breeds of cattle in the study area. Recent surveys showed that one and two breeds of cattle are kept in the Mambwe and Faro et Déo districts, respectively [44, 45]. The model incorporated the different breeds of cattle and heterogeneities in AAT risk within the Faro et Déo study area. Annual rates of mortality and offtake were calculated for the different age-sex classes to develop annual projections of herd growth. Unlike previous models, this model was stochastic to incorporate variability in cattle type and productivity, and uncertainty and variability surrounding estimates of AAT frequency and impact.

#### Model parameters

A comprehensive literature review was conducted to identify all of the relevant parameters for the cattle system and select appropriate values. Relevant observational, experimental infection and field studies of AAT, entomological surveys and studies of productivity of cattle were used to parametrize the model. Also, data from official sources (MSEG and TTCU) and a field study comprising interviews of cattle owners in the area were utilised appropriately [44, 45]. The main parameters used in the model are presented in Table 2. A full description of the parameter values used within the two case studies along with their source is provided in Additional file 1: market prices for inputs and outputs (Additional file 1: Tables S1 and S2), production parameters (Additional file 1: Table S3), herd management parameters (Additional file 1: Table S4), and AAT-related parameters (Additional file 1: Tables S5 and S6).

**Table 2:** Main parameters used in the bio-economic herd model. The indices i and b refer to the age-sex cattle class (calf, heifer, young male, cow, adult bull and adult ox) and breed respectively

Category	Notation	Parameter
Production	*m*_*b*_	Milk yield for breed *b* (kg per lactation)
	*l*_*b*_	Lactation length in breed *b* (days)
	*c*_*b*_	Annual calving rate in breed *b*
	*w*_*i,b*_	Live-weight of animals in class *i* and breed *b*, (kg)
Herd management	*o*_*i*_	Annual offtake rate of animals in class *i*
	*D*	Draught yield of oxen (days per year)
	*V*	Proportion of AAT deaths salvaged
Impact of AAT on productivity	*r*_*l*_	% reduction of milk production in animals affected by AAT
	*r*_*f*_	% reduction in fertility in animals affected by AAT
	*r*_*w*_	% reduction in live-weight in animals affected by AAT
	*r*_*d*_	% reduction in draught power in animals affected by AAT
Incidence and mortality	*T*	Proportion of AAT cases successfully treated
	*δ*_*s*_	Duration of symptoms when treatment succeeds, in days
	*δ*_*f*_	Duration of symptoms when treatment fails (days)
	*f*_*i*_	Fatality rate of AAT when treatment fails in animals of class *i*
	*μ*_*i*_	Baseline mortality in animals of class *i*, i.e. when AAT incidence = 0

#### Model analysis

**Fig. 2:**
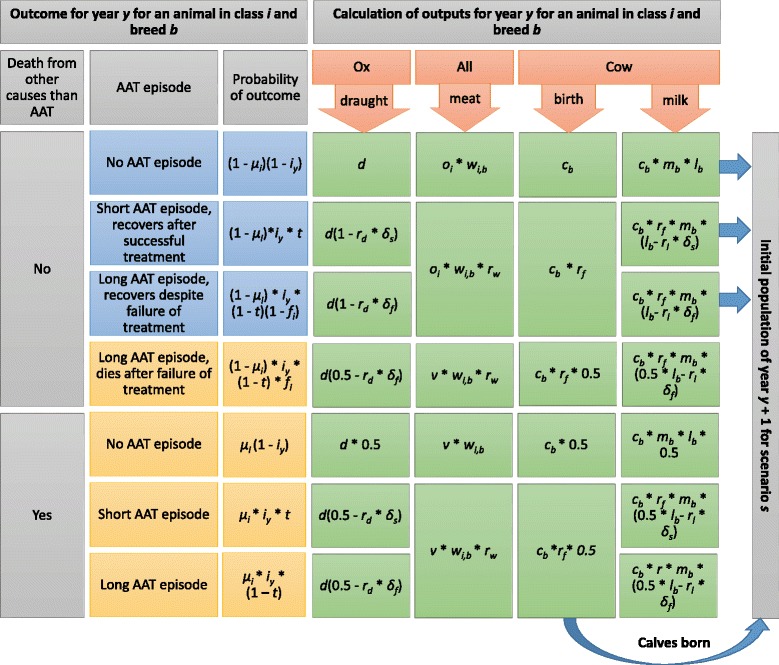
Infection outcomes and associated production outputs considered in the cost-benefit analysis, calculated at animal-level a given breed (b) and age-sex class of the animal (i). The parameter notations refer to those presented in Table 2

Census data obtained from the MSEG and the TTCU was used to define the initial herd structure (Additional file 1: Table S7). For each year *Y* of the simulation of a Scenario *S*, distributions of annual incidence determined the probability that each animal experienced an AAT event. Infection sequelae regarding the duration of illness and mortality were established for each affected animal to calculate the impact of AAT on its production during year *y* (Fig. 2). The total value of the herd production *P*_*Y,S*_ was calculated as the sum of the market values of the milk production, meat production and draught output of each animal. It was assumed that oxen and cows which died would do so halfway through their annual production cycle and that animals bred for meat production would die before they were slaughtered and have salvage value only (see Additional file 1: Table S8 for a full list of assumptions). The total value of the production inputs *I*_*Y,S*_ was calculated as the sum of the market values of the inputs related to AAT control (preventive trypanocides, curative trypanocides and insecticides) and the other inputs (non-AAT related rearing costs). Each animal with an AAT episode that year incurred one additional trypanocide dose. It was also assumed that farmers would cease the use of trypanocide prophylaxis and ITC once AAT was suppressed. Extra revenue was calculated as the total discounted value of cattle production *P*_*Y,0*_ under the baseline Scenarios (C0, Z0) for year *Y* subtracted from the total discounted value of cattle production *P*_*Y,S*_ under each control Scenario (C1 and 2, Z1 to Z4) for the same year *Y*. The difference between the total herd value under the baseline and control Scenarios was calculated at the end of the 10-year projection period (*H*_*10,S*_ - *H*_*10,0*_). The reduction in the total value of salvaged meat and the rearing costs of the additional cattle were incorporated in the additional costs. We assumed that all cattle products could be marketed and prices would remain inelastic under the different control Scenarios.

#### Model environment

The herd model was developed in R version 3.3.0 [46] using the packages *mc2d*, *psychometric* and *msm*. The additional costs linked to the control interventions were calculated in an Excel spreadsheet, using the cattle population size estimated in the herd model. Local prices were converted to US$ using average conversion rates from the year the data was collected (https://www.oanda.com/currency/average) and adjusted for the inflation rate accrued between the year the data was collected and the reference year of this study (2016) as described in Shaw et al. [25]. Annual rates of mortality and offtake were calculated for the different age-sex classes to develop annual projections of herd growth, and the model was run for 1000 iterations. The cost calculation was conducted in a deterministic manner.

#### Sensitivity analysis

Sensitivity analysis was used to examine the effect on the projects’ profitability of different variations from the initial study assumptions, reflecting potential departures from the processes and outcomes of the interventions. An increase in intervention costs (by 25% and 50%, respectively) a reduction in the technical intervention efficiency (final AAT incidence of 10%) and a reduction in the price of commodities due to an increase in supply was simulated. Estimates of the relative reductions in the prices of a commodity which might occur due to an increased supply and reductions in inputs were calculated on an annual basis using data from Kristjanson et al. [17]. The revenue obtained from milk, meat and draft in each scenario were then adjusted for purposes of sensitivity analysis.

## Results

### Net value of cattle production in the study areas

The annual net value of cattle under the baseline Scenarios was estimated to be 54.1 and 4.7 million US$ or 322 and 433 US$ per head in the Faro et Déo and Mambwe districts, respectively (Table 3). The majority of production revenue was estimated to derive from meat production in Faro et Déo, and milk production in Mambwe. Annual total spending on AAT control by farmers was estimated at 578,670 US$ (3.4 US$ per head) in Faro et Déo and 162,910 US$ (15 US$ per head) in Mambwe under the current control practices and AAT incidence. The majority of this was due to the purchase of preventative trypanocides. The cost of AAT-related inputs incurred per head of cattle was higher in Mambwe, due to the purchase of more trypanocides and the use of ITC, which is provided by the MSEG in Faro et Déo. Under the current AAT incidence levels, around 2% and 6% of the total herd were lost to AAT annually in Faro et Déo and Mambwe, respectively.

**Table 3:** Total net value of the cattle production (median, 5th and 95th percentiles) in the study areas for year 1 under the current AAT incidence (s = 0)

Output	Faro et Déo	Mambwe
Milk_1,0_ (10^6^ kg)	15.0 (14.7; 15.2)	2.2 (2.1; 2.3)
Milk_1,0_ (10^6^ US$)	4.5 (4.4; 4.6)	2.8 (2.6; 2.9)
Meat_1,0_ (10^6^ kg)	8.5 (8.3; 8.6)	0.20 (0.18; 0.24)
Meat_1,0_ (10^6^ US$)	10.0 (9.8; 10.2)	0.54 (0.50; 0.59)
Draught_1,0_ (10^6^ days)	2.2 (2.0; 2.3)	0.13 (0.12; 0.13)
Draught_1,0_ (10^6^ US$)	4.4 (4.1; 4.7)	0.64 (0.61; 0.66)
Total *P*_1,0_ (10^6^ US$)	18.9 (18.4; 19.3)	4.0 (3.3; 3.9)
Trypanocides_1,0_ (10^6^ US$)	0.58 (-0.57; -0.59)	-0.14 (-0.13; -0.15)
ITC_1,0_ (10^6^ US$)	–	-0.02
Other inputs_1,0_	-5.3	-0.50
Total *I*_1,0_ (10^6^ US$)	-5.9	-0.66
AAT deaths_1,0_ (head)	3515 (1498; 6348)	650 (251; 1237)
Total *H*_1,0_ (10^6^ US$)	41.2 (40.3; 41.9)	1.9 (1.8; 2.0)
Net value_1,0_ (10^6^ US$)	54.1 (52.9; 55.1)	4.7 (4.4; 4.9)

### Costs of the control programmes

The total control costs for Scenarios C1 and C2 were estimated at 1184 and 1768 US$ per km^2^ of infested area, respectively, using a discount rate of 5% (Table 4). The control costs for the Mambwe District ranged from 735 to 960 US$ per km^2^ of the infested area for the different Scenarios. The total control costs for Faro et Déo were higher as only a third of the district is infested and this includes some activities in the cleared plateau (such as monitoring).

**Table 4:** Detailed control costs incurred in Faro et Déo and Mambwe districts over the 10-year projection period, in USD per km^2^ infested except otherwise indicated

Study areas		Faro et Déo			Mambwe			
Field costs	SAT	na			506			
	ITC (excl. barrier)	151			na			
	Targets (excl. barrier)	365			448			
	ITC (for barrier) (US$ per km^2^ barrier)	618			735			
	Targets (for barrier) (US$ per km^2^ barrier)	1559			1792			
Monitoring costs (US$ per km^2^ district)		87			80			
Administrative overheads		196			166			
Scenarios		C0	C1	C2	Z1	Z2	Z3	Z4
Total discounted control costs (per km^2^ infested)^a^		680	1184	1768	735	898	799	960

^a^For Faro et Déo the total discounted control costs do not include the costs carried over from the baseline control programme

*Abbreviation*: *na* not applicable

### Results of the cost-benefit analysis

The benefit-cost ratios calculated for the different scenarios ranged between 2.0–4.5 (Table 5). The use of SAT in Mambwe as an elimination method yielded a higher BCR than the use of targets. The total discounted control costs and benefits for the 10-year period were estimated at 3.8 and 10.5 million US$ for Scenario Z3, respectively, which was the most cost-beneficial scenario for this study area. Total discounted costs and benefits were 6.5 and 29.6 million US$ for Scenario C1, which was the most profitable. The Zambian scenarios yielded NPVs between 4.8 and 6.8 million US$.

**Table 5:** Results of the cost-benefit analysis of the different scenarios

Scenario details		Total discounted benefits		Total discounted costs		Financial performance		
No.	Attack	Barrier	Costs saved (10^6^ US$)	Additional revenue (10^6^ US$)	Extra costs (10^6^ US$)	Revenue foregone (10^6^ US$)	BCR (5th; 95th percentile)	NPV 10^6^ US$ (5th; 95th percentile)
C1	Mixed	ITC	2.3	27.3	5.4	1.1	4.5 (4.4; 4.7)	17.8 (13.7; 21.9)
C2	Mixed	targets	2.3	27.3	6.6	1.1	3.8 (3.6; 4.0)	21.7 (18.0; 25.8)
Z1	Targets	ITC	0.8	8.8	3.7	0.5	2.3 (1.8; 2.7)	5.3 (3.3; 7.7)
Z2	Targets	targets	0.8	8.8	4.3	0.5	2.0 (1.6; 2.4)	4.8 (2.7; 7.1)
Z3	SAT	ITC	0.9	9.6	3.2	0.6	2.8 (2.3; 3.3)	6.8 (4.5; 9.4)
Z4	SAT	targets	0.9	9.6	3.7	0.6	2.5 (2.0; 2.9)	6.2 (3.9; 8.9)

### Sensitivity analysis

**Fig. 3:**
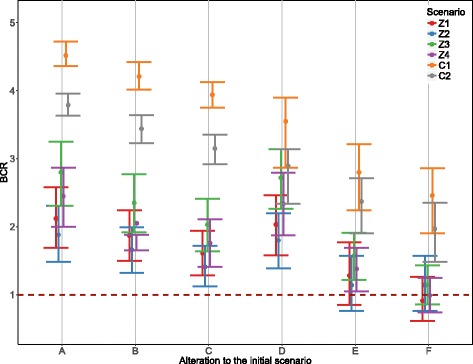
Benefit-cost ratios of the control scenarios under different perturbations to the initial parameters (median, 5th and 95th percentiles). Details of the perturbation to the scenarios: A (original scenario), B (+ 25% on intervention costs), C (+ 25*%* on intervention costs), D (final incidence 10%)

In response to increased commodities and the absence of any extreme market events, it was estimated that the annual price of meat, milk and draft in Cameroon could be reduced by up to 4.0%, 46.3% and 16.9% over the 10-year simulation period. In Zambia, estimates commodity prices were estimated to decrease by up to 42.6%, 73.3% and 48.1% for meat, milk and draft, respectively. This was simulated in Scenario E of the sensitivity analysis and remained profitable, with BCRs all higher than one. All other scenarios remained profitable (Fig. 3), except for Scenarios Z1 and Z4, when Scenario E was combined with a 50% increase in intervention costs. Overall, these results suggest the economic justification of control programmes is fairly robust and slight deviations from the planned processes or our model assumptions and outcomes regarding cost increases, or lower efficiency, are unlikely to significantly affect the profitability of the control programme.

## Discussion

AAT is deemed to be one of the main constraints to agricultural development in affected areas of sub-Saharan Africa [47] and has received increasing attention from international organisations and funding agencies in recent years. The proposed framework integrates an established decision support tool, aimed at optimising resource allocation regarding tsetse population management, with a detailed analysis of cattle productivity under different AAT control scenarios. Integration of these methods enables better-informed decisions regarding the pairing of priority tsetse-infested areas with a combination of control methods. The techniques are demonstrated in two example areas where the control effort depends on scarce national resources.

This study produced BCRs for AAT control interventions ranging between 2.0 and 4.6, indicating local tsetse elimination would be cost-beneficial in both settings. As farmers are currently paying for a large amount of trypanocide and insecticide doses, tsetse elimination would significantly reduce these costs and may slow down the development of trypanocide resistance in both study areas (a benefit not accounted for in this study). It is difficult to compare these results between settings due to the wide range of tsetse control methods which can be utilised, the varying impact that AAT can have on a community, the lack of economic analyses of AAT control programmes and the methodological variability of the existing ones [15]. They are within the range of values estimated by other authors; however, for example, a study in Senegal produced BCRs ranging between 0.98 and 4.26 [48]. In year 10, the estimated value of the production (milk, meat and draught power as well as herd value) was around 20% higher than the baseline in all scenarios. A herd model parametrized with data from Burkina Faso estimated that, compared to the reference herd, the monetary output from cattle production might increase by 12% to 74%, according to the different scenarios and the intensity of the production losses attributable to the AAT challenge [49]. This suggests that our assumptions are rather conservative; however, a large proportion of the production increase arises as a result of increasing herd size rather than increasing productivity, especially in Mambwe. Both study areas are situated on the wildlife-livestock interface, and livestock expansion is likely to be challenging in this regard due to competition for resources and space and lack of infrastructure [50, 51].

Net-present values for the study area in Cameroon were around 22 million US$, generating an additional 2000 US$ per km^2^. In Zambia, control of AAT was estimated to generate an additional 1061 to 1503 US$ per km^2^. In a previous survey of cattle-owning households across 17 study areas in five AAT-affected countries, communities in this region of Cameroon were concluded to have the highest vulnerability to AAT [14]. This was due to the high importance of cattle in this setting, a production system that includes large trypanosensitive transhumant herds, the reportedly high occurrence of treatment failure, constant AAT challenge and a lack of tsetse control in some areas [14]. This may explain the slightly higher profitability for Cameroon despite a much lower annual incidence, especially considering that cattle density in the area is 15.2 cattle/km^2^, which is closer to average cattle densities in tsetse-free sub-Saharan African regions [17]. Work by Shaw et al. [25] showed that the benefits of different control options are heavily influenced by cattle densities. Intervention costs in both study areas were comparable with other estimates [17, 23, 25, 33, 52]. The proportion of field costs to total costs in the two case studies varied between 67% and 89% according to the scenario considered. Other studies estimated the proportion of the field costs to be around 60% to 65% for similar techniques and an elimination campaign using the sterile insect technique [23, 48]; the lower proportion of field costs in the latter is likely related to the high overheads associated with the sterile insect technique.

The outputs from Tsetse Muse suggested that the techniques used in the attack phase of the different scenarios could eliminate tsetse in the study areas. However, successful examples of this are usually in isolated populations, and such populations are rare [15]. Indeed, previous control campaigns in Cameroon reportedly cleared areas of tsetse in the region, which however subsequently suffered from re-invasion or resurgence followed the cessation of control activities [27]. Previous elimination campaigns against savannah tsetse species using similar techniques were successful, for example in the Kwando-Zambezi tsetse belt encompassing the Western Province of Zambia and neighbouring countries, in Botswana and Zimbabwe [39, 53]. In this study, barriers would need to be maintained, and monitoring activities conducted continuously unless sequential elimination of the entire tsetse belt was achieved.

Cost-benefit studies should be supported by recent estimates of key parameters such as frequency of trypanosome infection and impact (e.g. duration of morbidity, mortality), livestock and tsetse demographics. Where available, this work used data collected from the specific study areas; however, there were key-data gaps. For example, the impact that AAT has on production parameters (milk, fertility, draught and meat yield) was extracted from literature reporting data collected in other settings and may vary according to factors such as breed, management system and trypanosome species present. A comprehensive review of the impacts was conducted, and this uncertainty was accounted for and incorporated in distributions. The initial mortality was high as the model assumed a high proportion of treatment failures. This was triangulated using several sources including interviews of farmers obtained from previous studies in the area, a previous study of resistance of trypanosome isolates and investigator experience in the area [6, 44, 45]. The mortality rate used in the model was extrapolated from an experimental field study of untreated zebu cattle in Kenya [54] but it is unlikely to give an accurate representation of field conditions in our study areas. However, this study was chosen as this breed (Orma Boran) exhibits some trypanotolerance, unlike breeds used in most experimental infection studies. Although Fulani, Gudali and Angoni breeds are considered trypanosensitive, it was assumed that many cattle were previously exposed to the disease (due to the high incidence in the study areas) and therefore they were not immunologically naïve [55, 56]. This is supported by interviews of cattle farmers in the study areas; less than 50% of farmers attributed cattle deaths in their herd to AAT in the previous 2 years, despite reporting AAT as a constant challenge, which is in agreement with the field experience of the investigators [44, 45]. Data on annual incidence rates were extracted from studies conducted several years ago and may not represent current levels. While this represents a limitation to this model, local experts suggested that the incidence rates are still high in both districts. Also, a recent study conducted in the District of Mayo Rey, close to the Faro et Déo district, estimated a prevalence of trypanosome infection in cattle of over 50% [57]. Although control activities have not reached the district of Mayo Rey, this suggests a high burden in this area of Cameroon; thus, the estimate that around 16% of transhumant cattle (entering the infested zone) are affected in a given year is reasonable. When studying the profitability of potential control projects, the benefits of eliminating tsetse increase over time, as the cattle population develops while the costs of maintaining barriers remain stable. Therefore, it is important to consider the long-term impact of these projects. However, the validity of model assumptions, such as inelasticity of market prices may be questionable over time. The choice of a 10-year projection is a reasonable compromise between these two constraints.

The current stocking rate of 2.4 head per km^2^ is very low in Mambwe District, Kristjanson et al. [17] estimated average cattle density in tsetse-free areas the same region and agro-ecological zone to be eight head per km^2^. This supports our assumption that the district grazing land can accommodate the projected increase in cattle numbers to a density of 6.0 head per km^2^ at the end of the 10-year period [58]. In Cameroon, cattle density was estimated to increase to 23.0 (C1 and 2), and 18.6 (C0) head per km^2^, which was considered feasible given that very few herds are present in the valley all year round. The benefits derived from increased productivity and costs saved as well as population growth. Besides economy-driven dynamics, important intangible dynamics, occurring over different timeframes, influence the efficacy and impact of such control interventions. Increasing herd sizes is a threat to wildlife due to competition for resources and transmission [50]. Integrated initiatives aiming at protecting biodiversity while improving livestock production are increasingly advocated, and environmental impact assessments are necessary [59–62]. Although Scenarios Z2 and Z4 (ITC barrier) yielded a higher BCR than Scenarios Z1 and Z3, respectively, experiments in comparable settings in Zimbabwe [53] found that a barrier of ITC in an area with eight to 12 cattle per km^2^ did not prevent reinvasion following removal of a target barrier. The reinvasion of tsetse into this area was attributed to the patchy distribution of cattle in some seasons. As the cattle density in Mambwe is around 2.4 cattle per km^2^ at present, an ITC barrier is unlikely to prevent reinvasion into the Mambwe District effectively. Hence, Scenarios Z1 and Z3 are not applicable at present but might be feasible in the future as cattle farming is a growing activity in the district. Economic analysis should be used as one of a range of tools, and the feasibility of planned interventions given current resources, infrastructure, technical expertise and the environmental and socio-political context should be considered [63, 64].

This study complements other research in the area of AAT control, aiming at informing optimal resource allocation and priority setting as well as estimations of costings [23, 65]; however, this study extends previous investigations by considering local dynamics and heterogeneities, including the consideration of different zones, breeds in Faro et Déo and farmer-based interventions. The study also summarises key factors that influence the impact of AAT on cattle production and profitability of control operations (Table 2 and Additional file 1), to advocate for appropriate data collection during control campaigns to allow *ex-ante* assessments. Existing control programmes provide a wealth of information for designing future control programmes. However, these are resources which are currently not fully exploited [15].

## Conclusions

As demonstrated in two study areas in Cameroon and Zambia, cost-benefit analysis can inform a priori the areas and control scenarios in which investment is likely to be most cost-beneficial. The data of this study have indicated that the elimination of tsetse populations from these study areas, particularly Faro et Déo will bring overall economic benefits for cattle farmers in these areas. The model allowed local heterogeneities, including different zones where cattle have different exposures to the disease to be considered. It is envisioned that the methodologies presented here can be adapted to other settings to aid the design and *ex*-*ante* assessments of future initiatives.

## Additional files

Additional file 1: Detailed data used to parameterize the bio-economic model and key assumptions [66–85]. (DOCX 33 kb)

## Abbreviations

AAT: animal African trypanosomiasis
BCR: benefit-cost ratio
HAT: human African trypanosomiasis
ITC: insecticide treatment of cattle
ITT: insecticide-treated targets
MSEG: Mission Spéciale d’Eradication des Glossines
NPV: net present value
PATTEC: Pan African Tsetse and Trypanosomiasis Eradication Campaign
SAT: sequential aerial spraying
T&T: tsetse and trypanosomiasis
TTCU: Tsetse and Trypanosomiasis Control Unit
