# Supplementary material for: Integrated cost-benefit analysis of tsetse control and herd productivity to inform control programs for animal African trypanosomiasis
Source: Parasit Vectors. 2018 Mar 7;11:154. doi: 10.1186/s13071-018-2679-x (PMC5842553; doi:10.1186/s13071-018-2679-x)
Supplement: Supplementary file 1 — Detailed data used to parameterize the bio-economic model and key assumptions [66–85]. (DOCX 33 kb) [file 13071_2018_2679_MOESM1_ESM.docx]

**Additional file 1: Detailed data used to parametrize the bio-economic model and key assumptions.**

Table S1: Current market prices used to calculate the control costs, according to study area: F: Faro et Déo; M: Mambwe; MSEG: Mission Spéciale d’Eradication des Glossines; TTCU: Tsetse and Trypanosomiasis Control Unit.

| **Parameter** | **Unit** | **Values** | **Reference** |
| --- | --- | --- | --- |
| **SAT-specific field costs** | | | |
| SAT field costs | USD per km^2^ | **M:** 506 | TTCU (aerial campaign in the Western Province of Zambia) |
| **ITC/trypanocide-specific field costs** | | | |
| Pour-on insecticide | USD per dose | **M:** 0.5 | TTCU |
|  |  | **FD:** 0.35 | MSEG |
| Field costs (transport, consumables) for ITC | USD per ITC head per year | **M:** 15.8 | TTCU |
|  |  | **FD**: 8.0 | MSEG |
| Trypanocide treatment by MSEG | USD per trypanocide dose per head | **FD**: 0.39 | MSEG |
| **ITT-specific field costs** | | | |
| Target | USD per unit | **M:** 3 | TTCU |
|  |  | **FD:** 3 | MSEG |
| Deltamethrin (20%) | USD per litre | **M:** 210 | TTCU |
|  |  | **FD:** 140 | MSEG |
| Attractants | USD per target per year | **M:** 10.6 | [23], TTCU |
|  |  | **FD:** 5 | MSEG |
| **General field costs** | | | |
| Staff salary and field allowances (executive staff) | USD per year per person | **M:** 15,000 | TTCU |
|  |  | **FD:** 25,258 | MSEG |
| Staff salary and field allowances (technicians) | USD per year per person | **M:** 9,600 | TTCU |
|  |  | **FD:** 7,081 | MSEG |
| Staff salary (casual workers) | USD per day per person | **M:** 14 | TTCU |
|  |  | **FD:** 10 | MSEG |
| 4x4 vehicle | USD per unit | 40,000 | TTCU, MSEG |
| Vehicles’ running costs (fuel, spares and maintenance) | USD per year per km^2^ infested | **M:** 8 | [23], TTCU |
|  |  | **FD:** 5.5 | MSEG |
| **Monitoring costs (per year)** | | | |
| T&T surveys and surveillance | USD per km^2^ | **M:** 26 | TTCU |
|  |  | **FD:** 16.4 | MSEG & Tsetse Muse |
| Environmental impact assessment and community awareness campaign | USD per km^2^ | **M:** 25 | TTCU |
|  |  | **FD:** 16.4 | MSEG & Tsetse Muse |

Table S2: Current market prices of relevant commodities in USD in Faro et Déo and Mambwe districts. F: Faro et Déo; M: Mambwe.

| **Parameter** | **Unit** | **Values** | **Reference** |
| --- | --- | --- | --- |
| Price of meat | USD per kg of live weight | **FD:** 1.2 | Local data collection |
|  |  | **M:** 3.0 |  |
| Price of meat from salvaged animals | USD per kg of live weight | **FD:** 0.6 | Local data collection |
|  |  | **M:** 1.5 |  |
| Price of milk | USD per kg | **FD:** 0.3 | Local data collection |
|  |  | **M:** 1.25 |  |
| Hire of pair of draught oxen | USD per day | **FD:** 6 | Local data collection |
|  |  | **M:** 10 | [66] |
| Cost of a cow | USD | **FD:** 350 | [67-69] |
|  |  | **M:** 190 | Local data collection |
| Cost of a calf | USD | **FD:** 60 | Local data collection, [67] |
|  |  | **M:** 95 | Local data collection |
| Cost of a heifer | USD | **FD:** 290 | [67-69] |
|  |  | **M:** 135 | Local data collection |
| Cost of a young male | USD | **FD:** 150 | Local data collection |
|  |  | **M:** 160 | Local data collection |
| Cost of an ox | USD | **FD:** 250 | Local data collection |
|  |  | **M:** 225 | Local data collection |
| Cost of a bull | USD | **FD:** 350 | Local data collection |
|  |  | **M:** 190 | Local data collection |
| Non-AAT-related rearing costs | Per head per year | **FD:** 32 | [70, 71] |
|  |  | **M:** 46.3 |  |

Table S3: Production parameters of cattle in Faro and Déo and Mambwe in the absence of AAT challenge, according to breed. F: Fulani; G: Gudali; A: Angoni; s.d.: standard deviation.

| **Parameter** | **Unit** | **Distribution** | **Values** | **Ref** |
| --- | --- | --- | --- | --- |
| Milk yield | Kg / lactation | Normal  (mean, s.d.) | **F:** 536 ± 100 | [72, 73] |
|  |  |  | **G:** 373 ± 100 |  |
|  |  |  | **A:** 990 ± 16 |  |
| Lactation length | Days | Normal | **F:** 175 ± 50 |  |
|  |  |  | **G:** 140 ± 45 |  |
|  |  |  | **A:** 294 ± 23 |  |
| Age at first calving | Months | Point estimate | **F/G:** 48 | [74, 75] |
|  |  |  | **A:** 39 | [76] |
| Calving rate | Per year | BetaPert  (min, mode, max) | **F:** 0.59, 0.761, 0.78 | [32, 73, 76, 77] |
|  |  |  | **G:** 0.67, 0.75, 0.78 |  |
|  |  |  | **A:** 0.44, 0.63, 0.85 |  |
| Live weight of cows | Kg | Uniform  (min, max) | **F:** 250, 380 | [78, 79] |
|  |  |  | **G:** 300, 410 |  |
|  |  |  | **A:** 250, 450 |  |
| Live weight of calves | Kg | Uniform  (min, max) | **F:** 22.3, 120.9 | [72, 80] |
|  |  |  | **G:** 22.5, 150 |  |
|  |  |  | **A:** 23, 147 |  |
| Live weight of heifers | Kg | Uniform  (min, max) | **F:** 114, 320 | [78, 79] |
|  |  |  | **G:** 159, 330 |  |
|  |  |  | **A:** 147, 450 |  |
| Live weight of young males | Kg | Uniform  (min, max) | **F:** 120, 350 |  |
|  |  |  | **G:** 150, 390 |  |
|  |  |  | **A:** 147, 300 |  |
| Live weight of oxen | Kg | Uniform  (min, max) | **F:** 350, 425 |  |
|  |  |  | **G:** 453 ± 50 |  |
|  |  |  | **A:** 300, 340 |  |
| Live weight of bulls | Kg | Uniform  (min, max) | **F:** 350, 665 |  |
|  |  |  | **G:** 395, 660 |  |
|  |  |  | **A:** 350, 570 |  |

Table S4: Herd management parameters for herds in the two study areas. F: Faro et Déo; M: Mambwe; s.d.: standard deviation.

| **Parameter** | **Unit** | **Distribution** | **Values** | **Ref** |
| --- | --- | --- | --- | --- |
| Offtake rate of cows | Per year | BetaPert(min, mode, max) | **FD:** 0.05, 0.113, 0.2 | [74] |
|  |  | Point estimate | **M:** 0 | Local data collection |
| Offtake rate of heifers | Per year | Point estimate | **FD:** 0.05, 0.113, 0.2 | [74] |
|  |  |  | **M:** 0.05 | Local data collection |
| Offtake rate of young males | Per year | BetaPert(min, mode, max) | **FD:** 0.05 | [81] |
|  |  |  | **M:** 0.1, 0.2, 0.3 | Local data collection |
| Offtake rate of oxen | Per year | Normal(mean, s.d.) | **FD:** 0.29 ± 0.05 | [81] |
|  |  | BetaPert(min, mode, max) | **M:** 0.1, 0.2, 0.3 | Local data collection |
| Offtake rate of bulls | Per year | Normal(mean, s.d.) | **FD:** 0.37 ± 0.05 | [81] |
|  |  |  | **M:** 0.5 ± 0.05 | Local data collection |
| Number of days worked (draught oxen) | Days / year | Normal(mean, s.d.) | 190 ± 19 | Local data collection¸ [82] |
| Proportion of AAT deaths salvaged | N/A | Point estimate | 0.33 | Local data collection |
| Proportion of males castrated | N/A | Point estimate | 0.5 | Local data collection |

Table S5: Impact of Animal African Trypanosomiasis on productivity and farmer based control-costs. F: Faro et Déo; M: Mambwe; s.d.: standard deviation.

| **Parameter** | **Unit** | **Distribution** | **Values** | **Ref** |
| --- | --- | --- | --- | --- |
| Reduction of milk production in animals affected by AAT | N/A | BetaPert  (min,mode,max) | 0.02, 0.15, 0.26 | [3, 83] |
| Reduction in fertility in animals affected by AAT | N/A | Uniform  (min, max) | 0.11, 0.22 | [3] |
| Reduction in live weight in animals affected by AAT | N/A | BetaPert  (min,mode,max) | 0.03, 0.08, 0.15 | [17] |
| Reduction in draught power in animals affected by AAT | N/A | BetaPert (min,mode,max) | 0.22, 0.38, 0.45 | [3] |
| Number of prophylactic doses of trypanocide | per animal per year | Point estimate | **FD:** 2 | Local data collection |
|  |  |  | **M:** 4 |  |
| Farmer-purchased trypanocide cost | USD per dose | BetaPert  (min,mode,max) | **FD:** 0.5, 0.8, 1 | [44] |
|  |  |  | **M:** 0.94, 2.5, 2.81 | [45] |
| Number of farmer-purchased doses of insecticide | per animal per year | Point estimate | **FD:** 0 | Local data collection |
|  |  |  | **M:** 3 |  |
| Farmer-purchased insecticide cost | USD per dose | Normal  (mean, s.d.) | **M:** 0.51 ± 0.05 | Local data collection |

Table S6: Parameters related to the incidence of Animal African Trypanosomiasis and mortality in Faro et Déo and Mambwe districts. F: Faro et Déo; M: Mambwe; s.d.: standard deviation.

| **Parameter** | **Unit** | **Distribution** | **Values** | **Ref** |
| --- | --- | --- | --- | --- |
| Current incidence sedentary valley Cameroon | N/A | Beta(a,b) | 4, 58 | [6] |
| Current incidence sedentary valley Cameroon | N/A | Beta(a,b) | 33, 29 |  |
| Current incidence transhumant herds Cameroon | N/A | Beta(a,b) | 94, 319 | [6, 38] |
| Current incidence Mambwe | N/A | Uniform(min, max) | 0.72, 1 | [32, 40] |
| Proportion of AAT cases successfully treated | N/A | Uniform(min, max) | **FD:** 0.36, 0.66 | [6, 84] |
|  |  |  | **M:** 0.51, 0.79 | [32] |
| Duration of symptoms when treatment succeeds | Days | BetaPert(min,mode,max) | 7, 14, 28 | [56] |
| Duration of symptoms when treatment fails / without treatment | Days | BetaPert(min,mode,max) | 42, 86, 242 |  |
| Fatality rate of AAT when treatment fails in cattle > 1 y | N/A | Uniform(min, max) | 0, 0.35 | [54, 56] |
| Fatality rate of AAT when treatment fails in cattle < 1 y | N/A | Uniform(min, max) | 0, 0.71 |  |
| Baseline mortality in adults (AAT incidence = 0) | N/A | Normal(mean ± s.d.) | **FD:** 0.034 ± 0.01 | [32, 69] |
|  | N/A |  | **M:** 0.06 ± 0.015 |  |
| Baseline mortality in young cattle (AAT incidence = 0) | N/A | Normal(mean ± s.d.) | **FD:** 0.046 ± 0.015 | [32, 72] |
|  | N/A |  | **M:** 0.097 ± 0.031 |  |
| Baseline mortality in calves (AAT incidence = 0) | N/A | Uniform(min, max) | **FD:** 0.07, 0.17 | [32, 67, 69] |
|  | N/A | Normal(mean ± s.d.) | **M:** 0.084 ± 0.026 |  |

Table S7: Current cattle population in Faro et Déo and Mambwe districts according to the 2015 census data obtained from the relevant Veterinary Services.

| **Cattle type** | **Faro et Déo** | **Mambwe** |
| --- | --- | --- |
| Cows | 42,059 | 4,328 |
| Heifers | 23,253 | 1,787 |
| Calves | 21,806 | 2,261 |
| Oxen | 17,466 | 816 |
| Bulls | 26,146 | 209 |
| Young males | 36,273 | 1,411 |
| **Total** | **168,003** | **10,812** |
| **Cattle density** | **15.2 head/km^2^** | **2.4 head/km^2^** |

*Table S8: List of key-assumptions used in the bio-economic herd model. The impact of the main assumptions on the financial viability of the control options were investigated using sensitivity analysis.*

| **Variables** | **Assumptions** | **Ref** |
| --- | --- | --- |
| AAT burden | | |
| **AAT Incidence** |  |  |
| Pre-elimination | Incidence will remain constant if no intervention | [6, 32, 40] |
| Post-elimination* | Elimination (annual incidence ≤5%) is achieved using the selected tools | [36, 85] |
| Age-sex class | All animals assumed to have the same annual risk of AAT | Structural assumption |
| **Mortality** |  |  |
| Adults | Mortality assumed to be slightly lower than experimental studies where cattle are completely naïve | [44, 45, 55, 56] |
| Calves | Mortality higher as assumed to be immunologically naïve | [53, 55] |
| Inputs | | |
| **Trypanocides** |  |  |
| Pre-elimination | All farmers use prophylactic trypanocides in both study areas: FD = 2 doses per animal per year, M = 4 | Local data collection |
| Post-elimination | All farmers would cease the use of prophylactic trypanocides immediately after elimination is achieved | [17] |
| **Other** |  |  |
| Rearing costs | Assume that rearing costs remain constant for the duration of the 10-year period | Local data collection |
| Outputs | | |
| **Draft production** |  |  |
| Age-sex class | All castrated adult males used for draft and 50% of males castrated | Local data collection |
| Mortality | Oxen assumed to have died half-way through the production year and therefore days-worked reduced by 50% | Structural assumption |
| **Milk** |  |  |
| Healthy cows | One lactation during a production-year. Calving is all year round | Structural assumption |
| Mortality | Cows that die are assumed to die half-way through the production cycle, therefore give half a lactation. | Structural assumption |
| **Calving** |  |  |
| Mortality | If a cow which dies was due to calve that year, there is a 50% probability they die before they calve. | Structural assumption |
| **Meat** |  |  |
| Slaughter | Assume all animals are slaughtered at the end of the production year. | Structural assumption |
| Mortality | Assume that all animals which die can have salvage value only. | Local data collection |
| **Prices of outputs** | | |
| Marketing of products | All products can be marketed. | Structural assumption |
| Prices* | Prices are inelastic and do not change due to increased productivity. | Structural assumption |
| **Projected cattle population** | | |
| Estimated growth | Ecosystem can support the projected growth in cattle population | [17] |
